# Mechanical ventilation with high tidal volumes attenuates myocardial dysfunction by decreasing cardiac edema in a rat model of LPS-induced peritonitis

**DOI:** 10.1186/1465-9921-13-23

**Published:** 2012-03-20

**Authors:** Lonneke Smeding, Frans B Plötz, Regis R Lamberts, Willem J van der Laarse, Martin CJ Kneyber, AB Johan Groeneveld

**Affiliations:** 1 Department of Pediatric Intensive Care, VU university medical center, Amsterdam, The Netherlands; 2 Department of Physiology, VU university medical center, Van der Boechorststraat 7, Amsterdam, The Netherlands; 3 Department of Intensive Care, VU university medical center, Amsterdam, The Netherlands; 4 Institute for Cardiovascular Research, VU University Medical Center Amsterdam, Amsterdam, The Netherlands; 5 Department of Pediatrics, Tergooi Hospital, Blaricum, The Netherlands; 6 Department of Physiology, School of Medicines, University of Otago, Dunedin, New Zealand; 7 Department of Pediatric Intensive Care, Beatrix Childrens Hospital/University medical center Groningen, Groningen, The Netherlands

**Keywords:** Ventilator-induced lung injury, Endothelial permeability, Myocardial depression, Myocardial edema

## Abstract

**Background:**

Injurious mechanical ventilation (MV) may augment organ injury remote from the lungs. During sepsis, myocardial dysfunction is common and increased endothelial activation and permeability can cause myocardial edema, which may, among other factors, hamper myocardial function. We investigated the effects of MV with injuriously high tidal volumes on the myocardium in an animal model of sepsis.

**Methods:**

Normal rats and intraperitoneal (i.p.) lipopolysaccharide (LPS)-treated rats were ventilated with low (6 ml/kg) and high (19 ml/kg) tidal volumes (Vt) under general anesthesia. Non-ventilated animals served as controls. Mean arterial pressure (MAP), central venous pressure (CVP), cardiac output (CO) and pulmonary plateau pressure (P_plat_) were measured. *Ex vivo *myocardial function was measured in isolated Langendorff-perfused hearts. Cardiac expression of endothelial vascular cell adhesion molecule (VCAM)-1 and edema were measured to evaluate endothelial inflammation and leakage.

**Results:**

MAP decreased after LPS-treatment and Vt-dependently, both independent of each other and with interaction. MV Vt-dependently increased CVP and Pplat and decreased CO. LPS-induced peritonitis decreased myocardial function *ex vivo *but MV attenuated systolic dysfunction Vt-dependently. Cardiac endothelial VCAM-1 expression was increased by LPS treatment independent of MV. Cardiac edema was lowered Vt-dependently by MV, particularly after LPS, and correlated inversely with systolic myocardial function parameters *ex vivo*.

**Conclusion:**

MV attenuated LPS-induced systolic myocardial dysfunction in a Vt-dependent manner. This was associated with a reduction in cardiac edema following a lower transmural coronary venous outflow pressure during LPS-induced coronary inflammation.

## Introduction

Septic patients often suffer from myocardial depression and acute lung injury, therefore requiring circulatory and ventilatory support [1]. The cause of the sepsis-induced myocardial dysfunction is multifactorial (for review see [2]) but endothelial activation is considered to be an important pathogenic mechanism [3]. Upon exposure to lipopolysaccharide (LPS) or tumor necrosis factor (TNF)-α, endothelium becomes activated as shown by expression of cell-adhesion molecules including myocardial vascular cell adhesion molecule (VCAM)-1 [4] immediately followed by an increase in endothelial permeability [5,6]. Endothelial permeability and subsequent leakage can cause myocardial edema, which indeed was shown after LPS infusion [7] and can hamper myocardial function [8].

Although the effect of mechanical ventilation (MV) on hemodynamics is well described [9], its role on sepsis-induced myocardial dysfunction is not well understood. Adverse effects of MV on myocardial contractility were previously suggested [10]. Recently, it has been suggested that alveolar injury caused by MV, also known as ventilator-induced lung injury (VILI), can induce a syndrome similar to sepsis [11,12], by inducing the release of inflammatory mediators from the lung. These mediators can increase microvascular permeability in lungs and extrapulmonary organs, like the kidney and potentially also in the heart, as a manifestation of remote VILI-induced organ injury in the course of biotrauma [12-14]. In line with this, exposure to perfusate from injuriously ventilated lungs induced increased microvascular permeability in uninjured lungs [15]. Also, a harmful stimulus in the lung, such as LPS, may induce pulmonary release of inflammatory mediators into the circulation that can increase VCAM-1 expression on liver vascular endothelial cells *in vitro *[16].

In this study we investigated the effect of MV with normal and injuriously high tidal volumes (Vt) on myocardial function during LPS-induced peritonitis. We hypothesized that MV with high Vt during LPS-induced peritonitis increases coronary VCAM-1 expression, thereby increasing cardiac edema and deteriorating myocardial function. Myocardial function was measured *ex vivo *in isolated hearts as myocardial function *in vivo *may be changed by differences in, amongst others, coronary flow, heart rate and loading due to MV [9].

## Methods

### Animal preparation and measurements

All experiments applied with the Guide for Care and Use for laboratory animals of the National Institute of Health and were approved by the Institutional Animal Care and Use Committee of the VU University Amsterdam. Male Wistar rats weighing 300 ± 20 g were anesthetized with 60 mg/kg pentobarbital (Nembutal, CEVA Santa Animale BV, Maassluis, The Netherlands) i.p. and 70 mg/kg ketamine (Alfasan, Woerden, The Netherlands) i.m. Anesthesia was maintained with pentobarbital (15 mg/kg) i.p. every 45 minutes and ketamine 20 mg/kg/h i.v. Paralysis was maintained with pancuronium (Organon, Oss, The Netherlands) 0.6 mg/kg/h i.v. to allow for high tidal volume mechanical ventilation. Rats were placed in supine position on a heating pad maintaining body temperature at 37°C. A tracheostomy was performed and a 14 Gauge canula was inserted into the trachea. During preparation, rats were ventilated with a tidal volume (Vt) of 6 ml/kg and 5 cm H2O positive end-expiratory pressure (PEEP) (Avea, CareFusion, Houten, The Netherlands). Catheters were inserted into the carotid artery and jugular vein for arterial blood sampling and continuous measurement of the mean arterial pressure (MAP) and central venous pressure (CVP) in mmHg after calibration and zeroing to atmospheric pressure. The femoral artery was catheterized with a thermistor from a pulmonary artery catheter to measure cardiac output (CO). CO was obtained every 30 minutes by averaging two successive thermodilution determinations (CO Computer, 9520A, Edwards Laboratory, Santa Ana, Ca, USA), for which 200 μl of cold saline was injected via the right jugular vein catheter as described previously [17]. Heart rate (HR) was measured by continuous electrocardiography (Viridia CMS 2000, Hewlett Packard, Boeblingen, Germany). Blood was taken every hour for gas analysis and blood samples were replaced by equal volumes of normal saline. Blood gas analysis was performed using a pH blood-gas analyzer (ABL 50; Radiometer, Copenhagen, Denmark). Partial pressure of arterial oxygen (PaO2, mmHg)/fraction of inspired oxygen (FIO2) ratios were calculated. Plateau pressure (Pplat) was taken from the ventilator.

### Experimental protocol

After preparation, hemodynamics were allowed to stabilize for 10 min after which base line values were established. Rats were randomized to non-LPS treated or LPS treated groups. The latter received LPS (7.5 mg/kg, LPS L2880, LPS from E. Coli 055:B5, Sigma-Aldrich) through an i.p. catheter. Five minutes after LPS infusion, the protocol was started and rats were randomly assigned to one of two ventilation strategies; ventilation with either low tidal volume (LTV; Vt 6 ml/kg, 5 cm H_2_O PEEP) or high tidal volume (Vt 19 ml/kg, 5 cm H_2_O PEEP). Thus four groups were studied; non-LPS treated (LTV-LPS, n = 7) and LPS-treated rats (LTV + LPS, n = 8) ventilated with low tidal volume and non-LPS treated (HTV-LPS, n = 11) and LPS-treated rats (HTV + LPS, n = 8) ventilated with high tidal volume. The F_I_O_2 _was set at 0.4 in both groups, ventilation rate was set to maintain normocapnia. MAP was maintained at a minimum of 60 mmHg by infusion of normal saline. Four hours after the start of the protocol hearts were rapidly dissected and mounted on an isolated Langendorff-perfused heart setup as previously described [18], to study myocardial function independent of loading condition.

### Myocardial function *ex vivo*

Myocardial function was measured *ex vivo*, as in this set-up it is possible to control loading, coronary pressure and heart rate, and to measure coronary flow. Next to the ventilated rats, non-ventilated rats, either non-LPS treated (control-LPS) or LPS-treated (control + LPS), were studied. The latter received LPS and were anesthetized and sacrificed after four hours. The groups contained 6 rats each, except for the control + LPS (n = 7) and HTV-LPS groups (n = 8). Briefly, the aorta of the isolated heart was cannulated and the heart was perfused with a modified Krebs-Henseleit solution at a constant coronary perfusion pressure of 80 mmHg at 37°C. The modified Krebs-Henseleit solution contained (in mM) 118.5 NaCl, 4.7 KCl, 1.4 CaCl_2_.2(H_2_O), 25 NaHCO_3_, 1.2 MgCl_2_, 1.2 KH_2_PO_4 _and 11 glucose and was equilibrated with 95% O_2 _and 5% CO_2 _at a pH of 7.4. Afferent coronary flow was measured with a flow meter (Transonic Systems Europe B.V., Maastricht, the Netherlands). Both right and left atria were removed and hearts were paced at 5 Hz with electrodes. A custom-made balloon was inserted in the left ventricle to measure isovolumic pressures with a catheter tip manometer system [18] and the heart was allowed to stabilize for 20 minutes. Ventricular volume at maximal pressure development (V_max_) was determined and balloons were adjusted to 85% of V_max_. Hearts were allowed to stabilize for another 10 min. After stabilization, myocardial function was measured by + dP/dtmax, -dP/dt_min_, systolic and diastolic pressure. Since measurements were performed at a fixed heart rate and preload, +dP/dt_max _and -dP/dt_min _can be regarded as indices of contractility and relaxation, respectively [19]. Developed pressure was calculated as left ventricular (LV) systolic pressure minus LV diastolic pressure. Tau, the time constant of relaxation was calculated by fitting pressure values of the descending part of a twitch with the formula a*(1-exp^t/tau^) + c. Fitting was started at 50% of developed pressure so that tau was calculated for late relaxation. After the protocol, lasting about 55 minutes, hearts were removed from the isolated Langendorff-perfused heart set-up, cut transversally in three sections, frozen in liquid N_2 _and stored at -80°C.

### Wet to dry weight ratios

Wet/dry ratios were used as a measure of edema. Immediately after the rats were sacrificed, middle right lung lob was taken and weighed, dried at 37°C and weighed again. Apical section of the heart was used to calculate cardiac wet/dry ratio. Frozen sections were weighed, freeze-dried and weighed again.

### VCAM-1 expression

Coronary VCAM-1 expression was determined by immunofluorescence microscopy. Cardiac cryosections (5 μm) were incubated with VCAM-1-antibody (1:40 sc-1504-R Santa Cruz, Calif, USA) at room temperature for 1 hour followed by anti-rabbit secondary antibody staining and subsequent rhodamine-conjugated wheat germ agglutinin (WGA for membranes, Molecular Probes Europe, Leiden, The Netherlands) and DAPI nucleus staining (Vectashield with DAPI, Vector Laboratories, Burlingame, CA, USA). Image acquisition was performed on a Zeiss Axiovert 200 M MarianasTM inverted microscope. Microscopy was performed with a 10× objective. The microscope, camera, and data were controlled by SlideBookTM software. In the images, regions of interest (the vascular endothelium) were selected by masking, previously described in detail [20]. Three masks were generated: the 1st by manually selecting the vasculature, the 2nd by automated selection of FITC fluorescence above background, indicating VCAM expression; and a 3 rd mask, which was the combination of mask 1 and 2, indicating VCAM expression in the vasculature. SlideBook™ software was used to determine the mean fluorescence intensity of the 3 rd mask in arbitrary units to indicate VCAM-1 expression. The surface positive for VCAM-1 was calculated as percentage of total vascular surface.

### Lung histology

After removal of the heart, the upper lobe of the left lung was isolated, 5% gelatin was instilled and lungs were frozen in liquid N2 and stored at -80°C. Pulmonary cryosections (5 μm) were cut, and gelatin was removed in warmed ethanol with consecutive concentrations ranging from 100-0%. HE-staining was performed. Per section, 5 photographs (9.4 μm^2^) were taken using a Leica DMRB microscope (Wetzlar, Germany). Intra-alveolar cells per photo were counted in a blinded fashion.

### Statistical analysis

Parameters were tested for normal distribution using Kolmogorov-Smirnov test. To analyze the effects of LPS, Vt and their interaction, non-parametric distributions were normalized by log-transformation or ranking and general estimated equations (GEE) were performed, taking repeated measures over time in the same rats and baseline values as covariates into account where appropriate. A statistically significant interaction implies that the effect of Vt dose over time differs among the non-LPS and LPS-treated rats. To assess the relation between myocardial function parameters and cardiac wet/dry weight ratios, non-normalized data were used and Spearman's rho correlation was calculated to correct for non-parametric distributions. Data are shown as mean ± SEM or median for scatter plot presentation. Exact P values are given if > 0.001. P < 0.05 was considered statistically significant.

## Results

### *In vivo *measurements

**Figure 1:**
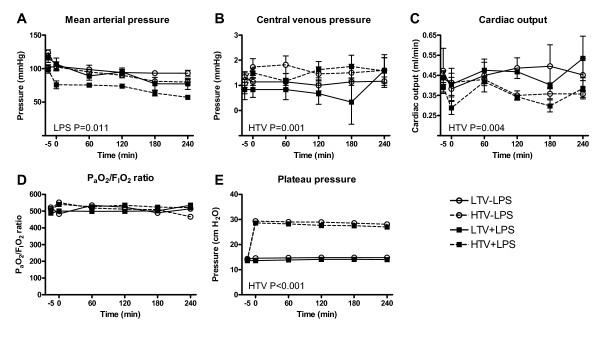
***In vivo *measurements in the course of time (LPS = lipopolysaccharide; LTV = low tidal volume, HTV = high tidal volume)**. A. Mean arterial pressure decreased by LPS treatment (P = 0.011). B. Central venous pressure increased by MV with high Vt (P = 0.001). C. Cardiac output decreased by MV with high Vt (P = 0.004). D Plateau pressure increased by MV with high Vt (P < 0.001) E. P_a_O_2_/F_I_O_2 _ratios were similar in the groups.

*In vivo *measurements are shown in Figure 1. Baseline characteristics were similar between the groups. MAP decreased in LPS-treated rats (P = 0.01) while CO and CVP were maintained. HR increased in non-LPS treated rats but not in LPS-treated rats (HR at t = -10 and t = 240; 224 ± 13 and 312 ± 14 beats/min versus 227 ± 12 and 267 ± 17 beats/min in non-LPS treated and LPS-treated rats respectively, both ventilation strategies taken together, P = 0.008). LPS treatment lowered blood pH (pH at t = -10 and t = 240; 7.38 ± 0.02 and 7.38 ± 0.02 versus 7.36 ± 0.01 and 7.25 ± 0.01, in non-LPS treated and LPS-treated rats respectively, both ventilation strategies taken together, P < 0.001), but had no effect on P_a_O_2_/F_I_O_2 _ratios. LPS-treated rats received more fluids than non-LPS treated rats (0.17 ± 0.07 mL in non-LPS treated rats versus 8.9 ± 1.4 in LPS-treated rats, P < 0.001). LPS treatment did not affect P_plat_. Increased Vt decreased MAP (P < 0.001) and more so after LPS treatment (P < 0.001 for interaction). Increased Vt increased CVP (P = 0.001; no interaction with LPS) and decreased CO (P = 0.004; no interaction with LPS). HR and fluid administration were not affected by Vt dose and no interaction with LPS was observed. Blood pH decreased with increasing Vt (pH at t = -10 and t = 240 7.39 ± 0.10 and 7.33 ± 0.03 versus 7.36 ± 0.10 and 7.28 ± 0.02 in LTV and HTV ventilation respectively, non-LPS treated and LPS-treated rats taken together, P = 0.003) but no interaction with LPS was observed. P_a_O_2_/F_I_O_2 _ratios were not affected by Vt dose or interaction with LPS. P_plat _increased with Vt (P < 0.001); no interaction with LPS was observed.

### Pulmonary injury

**Figure 2:**
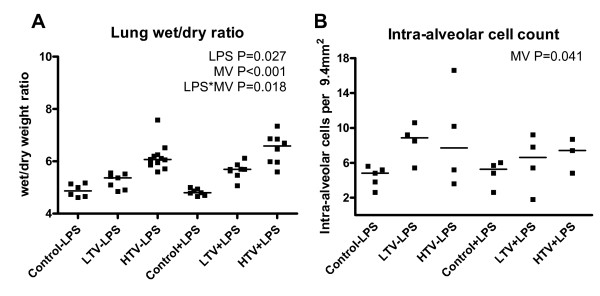
**Pulmonary injury**. A. Lung wet/dry ratio increased after LPS treatment (P = 0.027), Vt dependently (P < 0.001) and particularly when these were combined (P = 0.018) B. Intra-alveolar cell count increased after MV (P = 0.041).

Pulmonary wet/dry ratios were increased after LPS treatment (P = 0.027) and Vt dose dependently after MV (P < 0.001). A synergistic effect on pulmonary wet/dry ratios was observed (P = 0.018 for interaction, Figure 2a). Intra-alveolar cell count was increased upon MV (P = 0.041) but was not affected by LPS and no interaction was observed (Figure 2b).

### Myocardial function *ex vivo*

**Figure 3:**
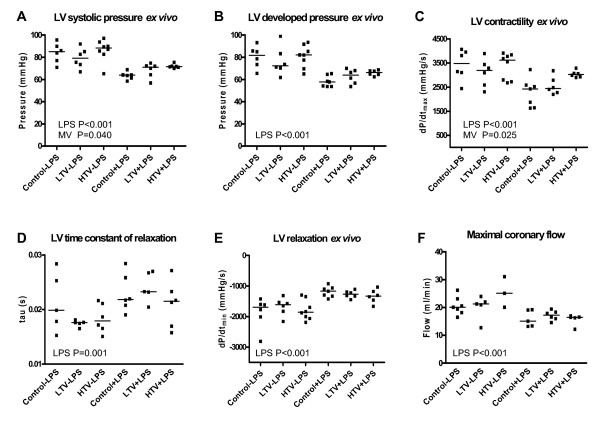
**Myocardial function *ex vivo *(LPS = lipopolysaccharide; LTV = low tidal volume, HTV = high tidal volume)**. A. Left ventricular systolic pressure decreased by LPS treatment (P < 0.001) and increased Vt-dependently (P = 0.04). B. Left ventricular developed pressure decreased by LPS treatment (P < 0.001). C. Left ventricular + dP/dt_max _decreased after LPS treatment (P < 0.001) and increased Vt-dependently (P = 0.025). D. Left ventricular time constant of relaxation tau increased after LPS treatment (P = 0.001.) E. Left ventricular - dP/dt_min _increased after LPS treatment (P < 0.001). F. Maximal coronary flow decreased after LPS treatment (P < 0.001).

Myocardial function data obtained *ex vivo *are shown in Figure 3. LPS-treated rats had a decreased myocardial function, as was shown by decreases in LV systolic pressure, LV developed pressure and + dP/dt_max _and increases in tau (P = 0.001) and -dP/dt_min _(P < 0.001). Maximal coronary flow was reduced in LPS-treated rats compared to non-LPS treated rats (P < 0.001), but not affected by MV. MV Vt dose-dependently improved myocardial contractile function, shown by increased LV systolic pressure (P = 0.04, no interaction with LPS) and + dP/dt_max _(P = 0.025; no interaction with LPS), but no effect on relaxation parameters tau and -dP/dt_min _were observed. LV end-diastolic pressure, and LV end-diastolic volume, estimated as V_max _were not affected by LPS, Vt dose or their interaction (data not shown).

### Cardiac VCAM-1 expression and wet/dry ratios

**Figure 4:**
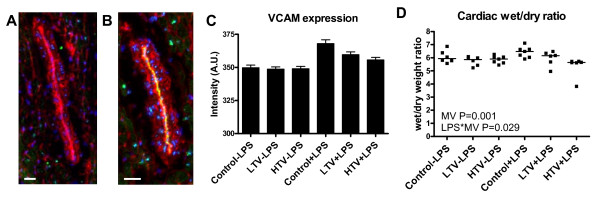
**Cardiac VCAM-1 expression and edema**. Immunofluorescent staining of VCAM-1 expression in A. non-LPS treated and B. LPS-treated rats (Bar = 25 μm). VCAM-1 expression (green), membranes (WGA, red) and nuclei (DAPI, blue) are visible. C. Fluorescence intensity in arbitrary units (A.U.) was increased after LPS treatment (P = 0.001). D Cardiac wet/dry ratio decreased Vt-dependently (P = 0.001) particularly after LPS treatment (P = 0.029).

LPS-treated rats had increased expression (P = 0.001) of coronary VCAM-1 and a greater percentage of myocardial vascular surface was positive for VCAM-1 expression than in non-LPS treated rats: (non-LPS treated rats 7.1 ± 0.8%, LPS-treated rats 10.7 ± 1.0%, P = 0.01, Figure 4a-c). MV did not affect VCAM-1 expression and no interaction was observed. Cardiac wet/dry ratios were similar between LPS treated and non-LPS treated rats (Figure 4d), however cardiac wet/dry ratios were decreased by increased Vt (P = 0.001) and this was more prominent in LPS-treated rats (P = 0.029 for interaction). Correlations were found between cardiac wet/dry ratios and LV developed pressure (r = -0.44, P = 0.006), LV systolic pressure (r = -0.46, P = 0.004), +dP/dt_max _(r = -0.36, P = 0.028), -dP/dt_min _(r = 0.35, P = 0.034) and tau (r = 0.40, P = 0.023).

## Discussion

In this study we hypothesized that MV with high Vt during LPS-induced peritonitis increases coronary VCAM-1 expression and cardiac edema and thereby aggravates LPS-induced myocardial dysfunction. In contrast to our hypothesis, MV with high Vt*in vivo *was associated with improved myocardial contractility *ex vivo*.

We used a LPS-induced peritonitis model as this model is reproducible [21] and targets, amongst others, the endothelium [11]. Indeed, LPS-induced peritonitis increased pulmonary vascular permeability, as suggested by pulmonary edema. In addition, Vt was set at 19 ml/kg to induce VILI, in accordance with the literature [22,23]. MV induced a further Vt-dose dependent increase in pulmonary edema and an increase in intra-alveolar cell count, despite unaltered P_a_O_2_/F_i_O_2 _ratios [22]. These characteristics of our model comply with early indicators of VILI [11]. Furthermore, LPS treatment decreased MAP and maintained CO and CVP without an increased heart rate, which indicates LPS-induced systemic vasodilatation and myocardial dysfunction, as cardiac work for a given preload decreased, and is consistent with previous observations [1]. As MAP decreased after LPS treatment, these rats received more fluids but this was not associated with an increase in cardiac edema. Finally, treatment with LPS characteristically reduced myocardial function *ex vivo *in line with previous observations [21,24]

MV with increasing Vt decreased cardiac output *in vivo*, however, it increased myocardial contractile function *ex vivo *as evidenced by an attenuated LPS-induced decrease in LV systolic pressure and + dP/dt_max_. Diastolic function, measured as -dP/dt_min _and tau, was not affected by MV. As coronary flow *ex vivo *was not affected by MV, the observed changes in myocardial function *ex vivo *after MV are not due to flow differences. We observed correlations between LV contractile and relaxation parameters and cardiac edema. As edema can hamper myocardial function [8], these correlations suggest that decreased cardiac edema was, at least in part, responsible for better myocardial contractile function in rats treated with LPS and MV with high Vt compared to rats treated with LPS and MV with low Vt.

Formation of edema depends on microvascular permeability through endothelial activation, and hydrostatic and osmotic pressure differences. VCAM-1 was chosen as parameter for endothelial activation since its coronary expression was shown to be upregulated 4 hours after LPS treatment [6,24]. Our study confirmed this, but the phenomenon was not affected by MV. The lack of effect of MV on VCAM-1 expression indicates that it is unlikely that the difference in edema among the modes of MV was caused by differences in microvascular permeability but rather resulted from differences in filtration pressure. We may speculate that a lower transmural coronary venous pressure during MV with high Vt was partly responsible for the decrease in edema, particularly during increased permeability in LPS-treated rats. Transmural pressure of the coronary veins, which is a driving force for cardiac edema, can be estimated from CVP-(mean airway pressure*pressure transmission). CVP increased by 1 mmHg during MV with high Vt. Mean airway pressure increased during high Vt ventilation by about 3.7 mmHg (5 cm H_2_O) as mean airway pressure depends on PEEP, which was similar between the two ventilated groups and P_plat_, which was higher during MV with high Vt. Airway pressure transmission is normally about 50%, but can decrease to 30% in diseased lungs due to increased stiffness [25]. These pressure differences result in a lower transmural pressure of the coronary veins during MV with high Vt, even in the case of a decreased airway pressure transmission, and this may have partially prevented edema formation. In any case, the hydrostatic and colloid osmotic pressure forces in the Langendorff-perfused heart set up were similar across groups, so that development of group differences in determinants of cardiac edema formation *ex vivo *can be excluded [26].

Although it is generally accepted that hydrostatic edema affects diastolic function [27], a decrease in myocardial edema after MV with high Vt did not attenuate LPS-induced diastolic dysfunction in our study. Hence, the decrease in diastolic function during LPS-induced peritonitis was only partly caused by increased permeability edema, thereby perhaps rendering diastolic function, in contrast to systolic function, relatively insensitive to attenuation of edema during MV with high Vt. In fact, most studies indicating edema-induced diastolic dysfunction were performed in otherwise healthy animals subjected to hydrostatic rather than increased permeability cardiac edema formation [8,27].

Cardiac output *in vivo *decreased with increasing Vt. In contrast, myocardial function *ex vivo *increased when animals were ventilated with increasing Vt. As HR did not change with increasing Vt*in vivo *and as myocardial function *ex vivo *was measured independent of changes in loading and, our data suggest that changes in cardiac output with high Vt*in vivo *were most likely caused by changes in loading conditions and not by changes in myocardial contractile function. Moreover, Gurkan et al. found increased inflammation in livers and kidneys but not in hearts of mice after acid aspiration and ventilation with high Vt [28]. Indeed, patients on MV often die from multiple organ failure and renal dysfunction may be more prevalent than cardiac dysfunction [12].

Limitations of our study include the absence of an intervention to show a direct relation between less myocardial edema and increased contractility *in vivo*. At the time the study was designed, a control group with injurious ventilation but unchanged airway pressure was not included. Moreover, in this study we measured myocardial function *ex vivo *as this provides the opportunity to measure function independent from differences in coronary flow, heart rate, loading and endocrine control. However, isolation of the heart from the whole animal may also be considered as a limitation as this takes the study further away from clinical relevance [29].

In conclusion, MV with high Vt lead to mild VILI but did not induce or aggravate myocardial dysfunction caused by LPS-induced peritonitis. Instead, LPS-induced myocardial dysfunction was attenuated in a Vt-dependent manner. Attenuation was most likely due to a decrease in cardiac edema as a result of a fall in transmural coronary venous pressure in face of increased endothelial activation and permeability, associated with coronary inflammation. This contrasts with the injurious effect of VILI on other remote organs like the kidneys [14].

## Competing interests

The authors declare that they have no competing interests.

## Authors' contributions

LS designed the experimental set-up, performed the experiments and drafted and revised the manuscript. RRL and WJL advised in the experimental design and revised the manuscript. FBP, MCJK and ABJG conceived the study, designed the experimental set-up and revised the manuscript. All authors read and approved the final manuscript.

## Note

These results have been presented in parts at the Annual congress of the European Society of Intensive Care Medicine (ESICM) 2009
